# 2,3-Dimethyl­anilinium chloride monohydrate

**DOI:** 10.1107/S1600536811043765

**Published:** 2011-10-29

**Authors:** Hossein Biglari Mazlaghani, Mohamad Reza Talei Bavil Olyai, Soudeh Hossein Zadeh, Behrouz Notash

**Affiliations:** a Department of Chemistry, Islamic Azad University, Karaj Branch, Karaj, Iran; b Department of Chemistry, Faculty of Science, Islamic Azad University, South Tehran Branch, Tehran, Iran; c Department of Chemistry, Shahid Beheshti University, G. C., Evin, Tehran 1983963113, Iran

## Abstract

The crystal structure of the title salt, C_8_H_12_N^+^·Cl^−^·H_2_O, consists of discrete organic cations, chloride anions and water mol­ecules which are connected by N—H⋯Cl, N—H⋯O and O—H⋯Cl hydrogen bonds. These inter­actions lead to the formation of layers lying parallel to the *ab* plane.

## Related literature

For related structures, see: Dai & Chen (2010 ▶); Abid *et al.* (2007 ▶). For hydrogen bonds, see: Steiner (2002 ▶); Jayaraman *et al.* (2002 ▶).
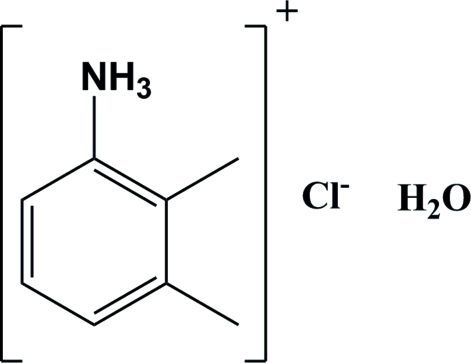


## Experimental

### 

#### Crystal data

C_8_H_12_N^+^·Cl^−^·H_2_O
*M* *_r_* = 175.65
Orthorhombic, 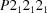

*a* = 7.4910 (15) Å
*b* = 7.5031 (15) Å
*c* = 17.430 (4) Å
*V* = 979.7 (4) Å^3^
*Z* = 4
Mo *K*α radiation
μ = 0.34 mm^−1^
*T* = 298 K
0.45 × 0.4 × 0.2 mm

#### Data collection

Stoe IPDS 2T diffractometer
4645 measured reflections
2616 independent reflections
2095 reflections with *I* > 2σ(*I*)
*R*_int_ = 0.031

#### Refinement

*R*[*F* ^2^ > 2σ(*F* ^2^)] = 0.038
*wR*(*F* ^2^) = 0.104
*S* = 1.03
2616 reflections
122 parameters
2 restraints
H atoms treated by a mixture of independent and constrained refinement
Δρ_max_ = 0.20 e Å^−3^
Δρ_min_ = −0.16 e Å^−3^
Absolute structure: Flack (1983 ▶), with 1088 Friedel pairs
Flack parameter: 0.13 (9)

### 

Data collection: *X-AREA* (Stoe & Cie, 2005 ▶); cell refinement: *X-AREA*; data reduction: *X-RED32* (Stoe & Cie, 2005 ▶); program(s) used to solve structure: *SHELXS97* (Sheldrick, 2008 ▶); program(s) used to refine structure: *SHELXL97* (Sheldrick, 2008 ▶); molecular graphics: *ORTEP-3 for Windows* (Farrugia, 1997 ▶); software used to prepare material for publication: *WinGX* (Farrugia, 1999 ▶).

## Supplementary Material

Crystal structure: contains datablock(s) I, global. DOI: 10.1107/S1600536811043765/bt5667sup1.cif

Structure factors: contains datablock(s) I. DOI: 10.1107/S1600536811043765/bt5667Isup2.hkl

Supplementary material file. DOI: 10.1107/S1600536811043765/bt5667Isup3.cml

Additional supplementary materials:  crystallographic information; 3D view; checkCIF report

## Figures and Tables

**Table 1:** Hydrogen-bond geometry (Å, °)

*D*—H⋯*A*	*D*—H	H⋯*A*	*D*⋯*A*	*D*—H⋯*A*
N1—H1*A*⋯Cl1^i^	0.95 (2)	2.21 (2)	3.1581 (17)	172.6 (17)
N1—H1*B*⋯O1	0.89 (3)	1.83 (3)	2.711 (2)	170 (2)
N1—H1*C*⋯Cl1^ii^	0.80 (2)	2.41 (2)	3.1964 (17)	171 (2)
O1—H1*W*⋯Cl1^iii^	0.82 (2)	2.35 (2)	3.1578 (19)	169 (3)
O1—H2*W*⋯Cl1	0.82 (2)	2.39 (2)	3.1920 (18)	168 (4)

Symmetry codes: (i) 
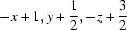
; (ii) 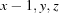; (iii) 
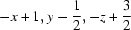
.

**Fig. 1.**
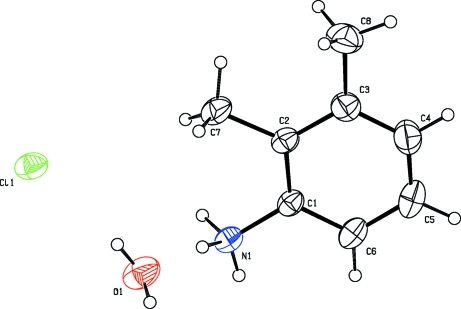
The asymmetric unit of title compound with displacement ellipsoids drawn at 50% probability level.

**Fig. 2.**
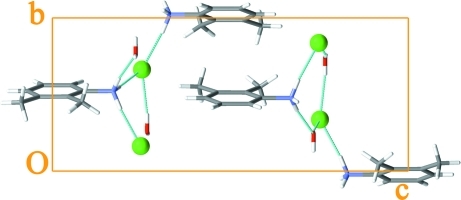
The packing diagram of the title compound showing the intermolecular N—H···O, N—H···Cl and O—H···Cl hydrogen bonds as dashed lines.

## supplementary crystallographic information

### Comment

Hydrogen bonding is of interest because of their prevalent occurrence in biological systems. Therefore, it is extremely useful to search simple molecules allowing to understanding the configuration and the function of some complex macromolecules. Furthermore, the hybrid materials are wealthy in H-bonds and they could be used to this outcome because of their capability emphasis in constructing sophisticated assemblies from isolated molecular or ionic building blocks due to its strength and directionality (Steiner, 2002; Jayaraman *et al.*, 2002).

As shown in Fig. 1, the asymmetric unit of (I) contains a 2,3-dimethylanilinium cation, a chloride anion and a water molecule. Packing diagram of the structure across the *a*-axis is shown in Fig. 2. It shows that each chloride anion connected to two 2,3-dimethylanilinium cations *via* N—H···Cl hydrogen bonds and two water molecules. The title complex is a crystalline hydrate containing one water of crystallization, where form layers through N—H···O and O—H···Cl hydrogen bonds (Table 1).

The C—NH_3_, 1.465 (2) Å distance in the organic cations are close to respect to the C—NH_3_, 1.459 (2) Å observed in the crystal structure of 2,3-dimethylaniliniumchloride (Dai & Chen, 2010). Moreover the organic group moiety geometrical features shows the C—C—C and C—C—N angles are in the range usually found for this compound (Abid *et al.*, 2007). The N—H···Cl and O—H···Cl hydrogen bond lengths are in the ranges of 2.21 (2)–2.41 (2) Å and 2.348 (18)–2.39 (2) Å, respectively. The organic species interact also with a strong N—H···O hydrogen bond with H···O separation of 1.83 (3) Å. Hydrogen bonds, electrostatic and van der Waals interactions participate to the cohesion of the three-dimensional network and add stability to this compound.

### Experimental

An initial solution of 2,3-dimetylaniline was made in 10 ml methanol. To a crystallizer vessel initial solution was added in a 1:1 molar ratio of concentrated hydrochloric acid dropwise. For salt formation partnership, the obtained solution was stirrer for 1 h and then gradually evaporated in room temperature. Crystals of the title salt were removed from the crystallizer vessel to yield colorless crystals of the title salt, suitable for X-ray analysis.

### Refinement

The H atoms of the protonated nitrogen and water molecule were found in difference Fourier map and refined isotropically. The water H atoms H1W, H2W were refined with distance restraints of O—H 0.844 (2), 0.860 (2) Å, respectively. The C—H protons were positioned geometrically and refined as riding atoms with C—H = 0.93 Å and *U*iso(H) = 1.2*U*eq(C) for aromatic C—H groups and C—H = 0.96 Å and *U*iso(H) = 1.5*Ueq*(C) for methyl group.

### Crystal data

**Table:**

C_8_H_12_N^+^·Cl^−^·H_2_O	*F*(000) = 376.0
*M**_r_* = 175.65	*D*_x_ = 1.191 Mg m^−^^3^
Orthorhombic, *P*2_1_2_1_2_1_	Mo *K*α radiation, λ = 0.71073 Å
Hall symbol: P 2ac 2ab	Cell parameters from 2616 reflections
*a* = 7.4910 (15) Å	θ = 2.3–29.2°
*b* = 7.5031 (15) Å	µ = 0.34 mm^−^^1^
*c* = 17.430 (4) Å	*T* = 298 K
*V* = 979.7 (4) Å^3^	Block, colourless
*Z* = 4	0.45 × 0.4 × 0.2 mm

### Data collection

**Table:**

Stoe IPDS 2T diffractometer	2095 reflections with *I* > 2σ(*I*)
Radiation source: fine-focus sealed tube	*R*_int_ = 0.031
graphite	θ_max_ = 29.2°, θ_min_ = 2.3°
Detector resolution: 0.15 pixels mm^-1^	*h* = −10→8
rotation method scans	*k* = −10→8
4645 measured reflections	*l* = −23→20
2616 independent reflections	

### Refinement

**Table:**

Refinement on *F*^2^	Secondary atom site location: difference Fourier map
Least-squares matrix: full	Hydrogen site location: inferred from neighbouring sites
*R*[*F*^2^ > 2σ(*F*^2^)] = 0.038	H atoms treated by a mixture of independent and constrained refinement
*wR*(*F*^2^) = 0.104	*w* = 1/[σ^2^(*F*_o_^2^) + (0.0572*P*)^2^ + 0.048*P*] where *P* = (*F*_o_^2^ + 2*F*_c_^2^)/3
*S* = 1.03	(Δ/σ)_max_ < 0.001
2616 reflections	Δρ_max_ = 0.20 e Å^−^^3^
122 parameters	Δρ_min_ = −0.16 e Å^−^^3^
2 restraints	Absolute structure: Flack (1983), with 1088 Friedel pairs
Primary atom site location: structure-invariant direct methods	Flack parameter: 0.13 (9)

### Special details

**Table:**

Geometry. All e.s.d.'s (except the e.s.d. in the dihedral angle between two l.s. planes) are estimated using the full covariance matrix. The cell e.s.d.'s are taken into account individually in the estimation of e.s.d.'s in distances, angles and torsion angles; correlations between e.s.d.'s in cell parameters are only used when they are defined by crystal symmetry. An approximate (isotropic) treatment of cell e.s.d.'s is used for estimating e.s.d.'s involving l.s. planes.
Refinement. Refinement of *F*^2^ against ALL reflections. The weighted *R*-factor *wR* and goodness of fit *S* are based on *F*^2^, conventional *R*-factors *R* are based on *F*, with *F* set to zero for negative *F*^2^. The threshold expression of *F*^2^ > σ(*F*^2^) is used only for calculating *R*-factors(gt) *etc*. and is not relevant to the choice of reflections for refinement. *R*-factors based on *F*^2^ are statistically about twice as large as those based on *F*, and *R*-factors based on ALL data will be even larger.

### Fractional atomic coordinates and isotropic or equivalent isotropic displacement parameters (Å^2^)

**Table:**

	*x*	*y*	*z*	*U*_iso_*/*U*_eq_	
O1	0.3476 (2)	0.2420 (2)	0.73382 (12)	0.0800 (5)	
Cl1	0.76090 (5)	0.33674 (6)	0.75167 (3)	0.06069 (15)	
N1	0.1163 (2)	0.4774 (2)	0.67017 (9)	0.0491 (3)	
C2	0.2579 (2)	0.5132 (2)	0.54351 (9)	0.0463 (3)	
C1	0.1057 (2)	0.4777 (2)	0.58624 (11)	0.0446 (4)	
C6	−0.0577 (3)	0.4395 (3)	0.55285 (12)	0.0621 (5)	
H6	−0.1572	0.4147	0.5829	0.075*	
C3	0.2418 (3)	0.5139 (2)	0.46329 (11)	0.0571 (4)	
C7	0.4338 (3)	0.5486 (4)	0.58231 (14)	0.0649 (5)	
H7A	0.4885	0.4375	0.5962	0.097*	
H7B	0.4144	0.6187	0.6276	0.097*	
H7C	0.5109	0.6123	0.5479	0.097*	
C5	−0.0691 (3)	0.4392 (4)	0.47360 (14)	0.0780 (7)	
H5	−0.1771	0.4134	0.4497	0.094*	
C8	0.4007 (4)	0.5513 (4)	0.41222 (15)	0.0825 (8)	
H8A	0.3627	0.5547	0.3596	0.124*	
H8B	0.4880	0.4588	0.4187	0.124*	
H8C	0.4522	0.6640	0.4259	0.124*	
C4	0.0778 (4)	0.4766 (3)	0.43060 (13)	0.0709 (6)	
H4	0.0676	0.4771	0.3774	0.085*	
H1C	0.021 (3)	0.451 (3)	0.6876 (13)	0.060 (6)*	
H1A	0.147 (3)	0.591 (3)	0.6905 (11)	0.052 (5)*	
H1B	0.198 (3)	0.400 (4)	0.6859 (13)	0.079 (8)*	
H1W	0.331 (4)	0.134 (2)	0.7337 (15)	0.088 (9)*	
H2W	0.456 (3)	0.257 (6)	0.7323 (19)	0.131 (13)*	

### Atomic displacement parameters (Å^2^)

**Table:**

	*U*^11^	*U*^22^	*U*^33^	*U*^12^	*U*^13^	*U*^23^
O1	0.0518 (8)	0.0643 (9)	0.1239 (15)	0.0014 (7)	−0.0103 (9)	0.0200 (10)
Cl1	0.0443 (2)	0.0566 (2)	0.0812 (3)	−0.00063 (17)	0.0040 (3)	0.0132 (2)
N1	0.0374 (7)	0.0507 (8)	0.0594 (9)	−0.0041 (6)	0.0022 (6)	−0.0012 (7)
C2	0.0419 (8)	0.0394 (7)	0.0576 (9)	−0.0017 (8)	−0.0004 (8)	0.0003 (6)
C1	0.0398 (7)	0.0380 (8)	0.0561 (9)	−0.0005 (7)	−0.0032 (7)	−0.0018 (7)
C6	0.0409 (8)	0.0708 (12)	0.0747 (12)	−0.0062 (8)	−0.0069 (8)	−0.0036 (10)
C3	0.0653 (11)	0.0487 (9)	0.0572 (10)	−0.0009 (11)	−0.0005 (10)	−0.0004 (7)
C7	0.0406 (9)	0.0825 (14)	0.0716 (12)	−0.0129 (9)	0.0035 (9)	−0.0003 (11)
C5	0.0602 (12)	0.0918 (17)	0.0822 (15)	−0.0073 (12)	−0.0260 (11)	−0.0093 (13)
C8	0.0918 (18)	0.0905 (18)	0.0652 (13)	−0.0146 (15)	0.0186 (13)	0.0035 (14)
C4	0.0820 (15)	0.0731 (14)	0.0576 (12)	−0.0029 (12)	−0.0156 (10)	−0.0011 (10)

### Geometric parameters (Å, °)

**Table:**

O1—H1W	0.820 (17)	C3—C4	1.383 (3)
O1—H2W	0.820 (18)	C3—C8	1.512 (3)
N1—C1	1.465 (2)	C7—H7A	0.9600
N1—H1C	0.80 (2)	C7—H7B	0.9600
N1—H1A	0.95 (2)	C7—H7C	0.9600
N1—H1B	0.89 (3)	C5—C4	1.361 (3)
C2—C1	1.388 (2)	C5—H5	0.9300
C2—C3	1.404 (2)	C8—H8A	0.9600
C2—C7	1.505 (3)	C8—H8B	0.9600
C1—C6	1.385 (2)	C8—H8C	0.9600
C6—C5	1.384 (3)	C4—H4	0.9300
C6—H6	0.9300		
			
H1W—O1—H2W	107 (4)	C2—C7—H7A	109.5
C1—N1—H1C	109.4 (16)	C2—C7—H7B	109.5
C1—N1—H1A	112.5 (11)	H7A—C7—H7B	109.5
H1C—N1—H1A	107 (2)	C2—C7—H7C	109.5
C1—N1—H1B	110.2 (15)	H7A—C7—H7C	109.5
H1C—N1—H1B	110 (2)	H7B—C7—H7C	109.5
H1A—N1—H1B	108 (2)	C4—C5—C6	120.0 (2)
C1—C2—C3	117.70 (16)	C4—C5—H5	120.0
C1—C2—C7	120.81 (15)	C6—C5—H5	120.0
C3—C2—C7	121.49 (16)	C3—C8—H8A	109.5
C6—C1—C2	122.69 (18)	C3—C8—H8B	109.5
C6—C1—N1	117.83 (17)	H8A—C8—H8B	109.5
C2—C1—N1	119.47 (15)	C3—C8—H8C	109.5
C5—C6—C1	118.3 (2)	H8A—C8—H8C	109.5
C5—C6—H6	120.8	H8B—C8—H8C	109.5
C1—C6—H6	120.8	C5—C4—C3	122.2 (2)
C4—C3—C2	119.08 (19)	C5—C4—H4	118.9
C4—C3—C8	119.6 (2)	C3—C4—H4	118.9
C2—C3—C8	121.29 (19)		
			
C3—C2—C1—C6	1.5 (3)	C7—C2—C3—C4	178.6 (2)
C7—C2—C1—C6	−178.15 (18)	C1—C2—C3—C8	−180.0 (2)
C3—C2—C1—N1	−179.41 (16)	C7—C2—C3—C8	−0.3 (3)
C7—C2—C1—N1	0.9 (3)	C1—C6—C5—C4	−0.3 (4)
C2—C1—C6—C5	−0.8 (3)	C6—C5—C4—C3	0.7 (4)
N1—C1—C6—C5	−179.9 (2)	C2—C3—C4—C5	0.0 (4)
C1—C2—C3—C4	−1.1 (3)	C8—C3—C4—C5	178.9 (2)

### Hydrogen-bond geometry (Å, °)

**Table:**

*D*—H···*A*	*D*—H	H···*A*	*D*···*A*	*D*—H···*A*
N1—H1A···Cl1^i^	0.95 (2)	2.21 (2)	3.1581 (17)	172.6 (17)
N1—H1B···O1	0.89 (3)	1.83 (3)	2.711 (2)	170 (2)
N1—H1C···Cl1^ii^	0.80 (2)	2.41 (2)	3.1964 (17)	171 (2)
O1—H1W···Cl1^iii^	0.82 (2)	2.35 (2)	3.1578 (19)	169 (3)
O1—H2W···Cl1	0.82 (2)	2.39 (2)	3.1920 (18)	168 (4)

Symmetry codes: (i) −*x*+1, *y*+1/2, −*z*+3/2; (ii) *x*−1, *y*, *z*; (iii) −*x*+1, *y*−1/2, −*z*+3/2.
